# Selenium seed priming enhanced the growth of salt-stressed *Brassica rapa* L. through improving plant nutrition and the antioxidant system

**DOI:** 10.3389/fpls.2022.1050359

**Published:** 2023-01-13

**Authors:** Saber Hussain, Shakil Ahmed, Waheed Akram, Guihua Li, Nasim Ahmad Yasin

**Affiliations:** ^1^ Guangdong Key Laboratory for New Technology Research of Vegetables/Vegetable Research Institute, Guangdong Academy of Agricultural Sciences, Guangzhou, China; ^2^ Institute of Botany, University of the Punjab, Lahore, Pakistan; ^3^ Department of Plant Pathology, Faculty of Agricultural Sciences, University of the Punjab, Lahore, Pakistan; ^4^ Senior Superintendent Gardner (SSG) Department, University of the Punjab, Lahore, Pakistan

**Keywords:** antioxidant, gene expression, NaCl, seed priming, Se, turnip

## Abstract

Various abiotic stresses may affect the germination, growth, and yield of direct-seeded vegetable crops. Seed priming with effective antioxidant mediators may alleviate these environmental stresses by maintaining uniformity in seed germination and improving the subsequent health of developing seedlings. Salt-induced stress has become a limiting factor for the successful cultivation of *Brassica rapa* L., especially in Southeast Asian countries. The present study was performed to elucidate the efficacy of seed priming using selenium (Se) in mitigating salt-induced oxidative stress in turnip crops by reducing the uptake of Na^+^. In this study, we administered three different levels of Se (Se-1, 75 μmol L^−1^; Se-2, 100 μmol L^−1^; and Se-3, 125 μmol L^−1^) alone or in combination with NaCl (200 mM). Conspicuously, salinity and Se-2 modulated the expression levels of the antioxidant genes, including catalase (*CAT*), peroxidase (*POD*), superoxide dismutase (*SOD*), and ascorbate peroxidase (*APX*). The upregulated expression of stress-responsive genes alleviated salt stress by scavenging the higher reactive oxygen species (ROS) level. The stress ameliorative potential of Se (Se-2 = 100 μmol L^−1^) enhanced the final seed germination percentage, photosynthetic content, and seedling biomass production up to 48%, 56%, and 51%, respectively, under stress. The advantageous effects of Se were attributed to the alleviation of salinity stress through the reduction of the levels of malondialdehyde (MDA), proline, and H_2_O_2_. Generally, treatment with Se-2 (100 μmo L^−1^) was more effective in enhancing the growth attributes of *B. rapa* compared to Se-1 (75 μmo L^−1^) and Se-3 (125 μmo L^−1^) under salt-stressed and non-stressed conditions. The findings of the current study advocate the application of the Se seed priming technique as an economical and eco-friendly approach for salt stress mitigation in crops grown under saline conditions.

## Introduction

Selenium (Se) is an essential micronutrient required for the normal growth of plants subjected to several abiotic and biotic stresses (Kaur et al., 2022). However, a higher amount of this element may have harmful effects on the physiochemical activities of plants (Shafiq et al., 2019). Selenium may enter the food chain through the consumption of plant-based food items (Feng et al., 2021). Hence, it is important to study its effects and outcomes in living organisms. In the case of plants, Se triggers various metabolic activities by assisting in the biosynthesis of selenoenzymes. Selenoenzymes act as antioxidants and safeguard cellular membranes through the detoxification of reactive oxygen species (ROS) (Abou El-hamd and Ahmed, 2021). Nevertheless, the pro-oxidant activity of a higher concentration of Se causes oxidative damage and reduces plant growth (Galić et al., 2021). Similarly, different types of this element also induce varying effects on different plant tissues (Zulfiqar et al., 2022). Sulfur (S) and Se use similar S-transporter pathways in plants and form selenomethionine (SeMet) or selenocysteine (SeCys) (Hossain et al., 2021). The methylated form of Se is non-toxic for various plants (Sarwar et al., 2020).

It is a universal truth that conventional breeding techniques, as well as genetic engineering, may help in the development of cultivars with good germination potential and yield. However, these breeding techniques are time-consuming, while genetic engineering has become a contentious approach (Johnson and Puthur, 2021). To overcome such difficulties, plant genes of the abiotic stress-tolerant Brassicaceae family have been characterized during the last three decades and are being further exploited for the development of varieties with higher productivity (Zhu et al., 2021). The cost of genetic engineering and conventional breeding procedures for the development of stress-tolerant cultivars is a significant constraint to the success of these approaches. Therefore, economical and eco-friendly techniques are necessary to raise successful crops under extreme environmental conditions. Seed priming using different elements, including phytohormones, acids, salts, vitamins, and minerals, may induce stress tolerance in developing seedlings to help combat biotic and abiotic stresses (Alves et al., 2021; Zaid et al., 2022). However, determining the appropriate concentration and priming duration of the nutri-priming agents is crucial in enhancing seed vigor and alleviating plant stress (Kumar et al., 2020). Therefore, it is mandatory to observe the prior optimization of nutrients such as Se before their use as priming elements. Researchers have confirmed the beneficial effects of Se as a priming agent, yet there is a dearth of information on the application of this dynamic nutrient in the case of turnip stress alleviation under saline regimes.

Turnip (*Brassica rapa*) is a common horticultural crop cultivated worldwide. The seeds of this crop are used for the extraction of cooking oil and biofuel (Cartea et al., 2019). In contrast, the foliage and root of *B. rapa* are used as a vegetable. Moreover, the glucosinolates isolated from this plant have tremendous medicinal value in the treatment of various types of cancer (Paul et al., 2019). Unfortunately, the continuously increasing salinity has reduced the yield of this crop in salt-affected areas of the world.

Salinity is a common cause of abiotic stress in plants, which may cause 65% yield loss in crop plants (Esmaeili et al., 2021). Higher salt accumulation in arable land impedes the physiological and biochemical activities of plants by inducing salinity stress (Fariduddin et al., 2019). The increased uptake and translocation of sodium ions (Na^+^) reduces the water uptake in plants, resulting in the induction of osmotic stress. Similarly, elevated levels of Na^+^ reduce the uptake of essential plant mineral cations such as calcium (Ca^2+^) and potassium (K^+^) (Kapadia et al., 2021). Decreased levels of Ca^2+^ and K^+^ cause the deterioration of cellular integrity and hamper plant growth (Alnusairi et al., 2021). In addition, higher levels of ROS induce oxidative damage in salt-stressed plants (Challabathula et al., 2022). Salinity stress spoils the photosynthetic machinery, impedes physiological progressions, including stomatal conductivity, and decreases the leaf area of plants (Ahanger et al., 2019).

Keeping in view the beneficial effects of Se seed priming in various crops, it was assumed that priming *B. rapa* seeds with this dynamic element could enhance seed germination and seedling growth by favoring the growth, related physiochemical activities, synthesis of photosynthetic pigments, and improvement of the antioxidant machinery under salt stress. The core objective of the current study was to unravel the potential application of Se seed priming in regulating *B. rapa* germination and seedling growth under saline conditions. The priming-induced modulations of the physiological and metabolic characteristics of *B. rapa* seeds and seedlings were also explored and compared.

## Methodology

### Seed priming and growth conditions

The present research work was carried out in a laboratory at the Institute of Botany, University of the Punjab, Lahore, Pakistan. The experiments had a completely randomized design (RCBD), which included eight treatments and five replications. The treatments used Se as the seed priming agent administered at three concentration levels—75 μmo L^−1^ (Se-1), 100 μmo L^−1^ (Se-2), and 125 μmo L^−1^ (Se-3)—and one level of salt stress given as 200 mM NaCl, in accordance with Sogoni et al. (2021). Distilled water was used as the control. Uniform, healthy-looking seeds of *B. rapa* var. Purple Top White Globe, obtained from Ayub Agricultural Research Institute (AARI), Faisalabad, Pakistan, with 10% initial moisture content (dry weight basis), were surface sterilized by dipping them in a 0.5% solution of sodium hypochlorite for 3 min. The sterilized seeds were subsequently washed meticulously with distilled water. For Se pretreatment, air-dried seeds were soaked in the respective Se solution (75, 100, or 125 µmo L^−1^) prepared by dissolving Na_2_SeO_3_ in distilled water. The glass flasks containing these seeds were placed in the dark overnight for 12 h at room temperature and aerated with an orbital shaker. The primed seeds were then removed from the flask and dried by placing them over two coats of sterile blotting papers. The seeds were then air-dried at room temperature. Afterward, nine seeds from each treatment were placed over two coats of sterile filter papers in 90-mm Petri plates. Subsequently, 10 ml NaCl solution (200 mM) was added to allocated Petri plates as the designated stress level, while distilled water was used in others. All Petri dishes were placed inside a growth chamber with a light intensity of 18 μmol photon m^−2^ s^−1^ (16-h light/8-h dark) at 25°C. Seeds showing at least ≥2 mm radical growth were considered germinated. Germination data were recorded after 5, 10, and 15 days.

The equation proposed by Ellis and Roberts (1981) was employed to analyze the germination percentage (final germination percentage, FGP) after 5, 10, and 15 days of sowing.

FGP = $\frac{\text{No}.\text{ of seeds germinated after }15\text{ days}}{\text{Total number of seeds}}$ × 100

The 15-day-old seedlings were then removed from the Petri plates, rinsed three times with distilled water, followed by surface drying by placing them over the blotting papers, and then immediately stored in liquid nitrogen for further examination.

### Estimation of biomass production

The seedling length was analyzed using a scale, while fresh weight was measured using an electrical weight balance.

### Quantification of total soluble sugar, total soluble protein, proline, and malondialdehyde contents

The content of total soluble sugar (TSS) in the seedlings was analyzed employing the anthrone technique developed by Irigoyen et al. (1992). Bradford’s (1976) technique was followed to calculate the content of total soluble protein (TSP), while proline content was quantified using the method of Bates et al. (1973). For the estimation of the malondialdehyde (MDA) level, the extent of lipid peroxidation was measured using thiobarbituric acid (TBA), as illustrated by Heath and Packer (1968).

### Estimation of chlorophyll content

The contents of chlorophyll a (Chl *a*), chlorophyll b (Chl *b*), carotenoids, and total chlorophyll from the prewashed fresh foliage samples were determined according to Lichtenthaler and Wellburn (1983). For this purpose, a 0.1-g shoot sample was homogenized with 20 ml of 80% acetone (*v*/*v*) and centrifuged at 10,000 × *g* for 3 min, then stored and covered on ice until use. Thereafter, the spectrophotometric value of the extract was observed at 663, 645, and 480 nm to calculate the Chl *a*, Chl *b*, and total chlorophyll contents, respectively. The carotenoids were extracted with 80% acetone and assessed according to Lichtenthaler (1987).

### Estimation of α-amylase activity

After 15 days of incubation, the crude extract from the treated seedlings was prepared according to Sottirattanapan et al. (2017) by centrifugation at 10,000 × *g* for 10 min at 4°C. The activity of α-amylase from the supernatant was quantified according to the 3,5-dinitrosalicylic acid technique of Miller (1959) by observing the absorbance at 540 nm.

### Quantification of Se content and Na uptake

The number of mineral nutrients, including Na and Se, was examined from the finely ground oven-dried plant samples. The Na content was estimated using a flame photometer (Baruah et al., 1998). The technique developed by Thimmaiah (1999) was used to estimate the Se content.

### Activity of antioxidant enzymes

A foliage sample (1 g) ground with liquid nitrogen was mixed with pre-chilled potassium phosphate buffer (50 mM) along with 1% (*w*/*v*) polyvinylpyrrolidone and 2 mM Na-EDTA (pH 7.0). The solution was centrifuged at 14,000 × *g* for 15 min at 4°C. The activities of antioxidant enzymes, including catalase (CAT), peroxidase (POD), and superoxide dismutase (SOD), were analyzed from the resulting supernatant using the methods of Chance and Maehly (1955); Aebi (1974), and Dhindsa et al. (1981), respectively.

### RNA extraction and gene expression

Another independent experiment was conducted in which the freshly detached shoot sample of 15-day-old Se-2-treated *B. rapa* plants was immediately stored in liquid nitrogen. For the extraction of total RNA, the TransZol Up reagent (TransGen Biotech, Beijing, China) was used. RNA quality and quantity were analyzed using the Nanodrop 2000 spectrophotometer (Thermo Fisher Scientific, Waltham, MA, USA). For complementary DNA (cDNA) production, 2 µg RNA was transferred into the TransScript One-Step gDNA Removal and cDNA Synthesis SuperMix kit according to the manufacturer’s instructions (TransGen Biotech, Beijing, China). Subsequently, a 1:10 dilution of cDNA was used for quantitative real-time PCR analysis with the TransStart Tip Green qPCR SuperMix (TransGen Biotech, Beijing, China) attached to a Roche LightCycler 480 thermal cycler instrument (384-well; Roche, Basel, Switzerland). The primers listed below were used to assess the expression levels of the *APX*, *SOD*, *POD*, and *CAT* genes (Zhang et al., 2020).

**Table:** 

Primer sequences (5′–3′)		
Genes	Forward	Reverse
*SOD*	TCACTGACAGCCAGATTCCTC	CCTGCGTTTCCAGTAGACAA
*CAT*	GCCGAACCCGAAAACAAA	GTCATCAAACATCCAGCACCA
*APX*	CCCATTCGGAACAATGAGGT	ACAGCCACAACACCAGCAAG
*POD*	CTCCCACCGTATCCTCG	CGTGCTGCTCAAAGTCG

### Statistical analysis

The data obtained were presented as the average of five replicates. The influence of the different treatments was evaluated using analysis of variance *via* the general linear model process of Statistical Analysis System (SAS Statistix 8.1 software). Tukey’s honestly significant difference test was used to analyze differences in the mean values of various treatments at a 5% significance level.

## Results

### Germination percentage and seedling growth

The results of the present study showed that, during seedling germination, salinity stress (200 mM NaCl) significantly inhibited *B. rapa* seed germination by 53% compared to seeds grown in the control condition (**Table 1**). However, Se pretreatment improved the FGP by 88% and 100% in the stressed and non-stressed conditions, respectively (**Table 1**). Of the three priming treatments, Se-2 (100 μmo L^−1^) and Se-3 (125 μmo L^−1^) were 100% more effective in restoring the FGP compared to Se-1 (75 μmo L^−1^), which restored the FGP by 88% after 15 days of germination. Se-primed seedlings grown without NaCl stress showed a relatively enhanced seedling length (58%) compared to the control, indicating the positive influence of Se (**Table 1**). Salinity stress inhibited seedling length by 45% and 30% compared to the control at 10 and 15 days, respectively (**Table 1**). Seed priming with Se-2 enhanced the length of *B. rapa* seedlings by 33% when grown under NaCl stress conditions.

**Table 1:** Influence of Se pretreatment on seed germination (in percent) and vegetative growth of *Brassica rapa* seedlings under saline conditions.

Treatments	FGP			Seedling length (cm)	Seedling fresh weight (g/plant)
	5 DAS (%)	10 DAS (%)	15 DAS (%)		
Control	30.55 ± 0.48f	100 ± 0.00a	100 ± 0.00a	8.94 ± 0.092b	0.87 ± 0.086a
NaCl	11.12 ± 0.25g	41.67 ± 0.40d	52.76 ± 0.21c	3.06 ± 0.092f	0.64 ± 0.05e
Se-1	44.45 ± 0.48e	86.12 ± 0.40bc	88.89 ± 0.41b	7.84 ± 0.23c	1.04 ± 0.05bc
Se-2	66.65 ± 0.28a	95.12 ± 0.25ab	100 ± 0.00a	13.02 ± 0.33a	1.66 ± 0.08a
Se-3	86.11 ± 0.62a	100 ± 0.00a	100 ± 0.00a	6.06 ± 0.12d	0.98 ± 0.07cd
NaCl+Se-1	55.56 ± 0.40d	77.76 ± 0.28c	88.89 ± 0.21b	5.6 ± 0.17de	0.96 ± 0.52cd
NaCl+Se-2	88.89 ± 0.25c	88.89 ± 0.25bc	100 ± 0.00a	9.7 ± 0.21b	1.2 ± 0.07b
NaCl+Se-3	77.78 ± 0.85b	94.45 ± 0.21b	100 ± 0.00a	4.7 ± 0.23e	0.83 ± 0.03d

Data presented are the mean ± SD of five replications. Different letters denote significant differences between treatments at p ≤ 0.05. NaCl = 200 mM NaCl; Se-1 = 75 μmo L^−1^ Se; Se-2 = 100 μmo L^−1^ Se; Se-3 = 125 μmo L^−1^ Se.

FGP, final germination percentage; DAS, days after sowing; C, control.

### Biomass production

Salinity stress reduced the seedling biomass accumulation by 55% compared with non-NaCl stress (**Table 1**). When Se-primed seeds were grown under non-stressed conditions, these seedlings exhibited 52% and 18% significantly higher biomass production compared to the salt-stressed and non-stressed conditions, respectively (**Table 1**).

### Chlorophyll content

Selenium seed priming, particularly Se-2 (100 μmo L^−1^), showed beneficial effects on the biosynthesis of photosynthetic contents, with increases in Chl *a*, Chl *b*, carotenoids, and total chlorophyll of 28%, 34%, 62%, and 37%, respectively, in *B. rapa* seedlings developing under normal conditions (**Table 2**). Se-2-treated seedlings exhibited a significantly elevated photosynthetic content compared to seedlings grown in the NaCl-spiked and control conditions (**Table 2**).

**Table 2:** Influence of Se pretreatment on the photosynthetic pigments and total soluble protein of *Brassica rapa* seedlings under saline conditions.

Treatments	Chlorophyll *a* (mg g^−1^ FW)	Chlorophyll *b* (mg g^−1^ FW)	Total chlorophyll (mg g^−1^ FW)	Total soluble protein (mg g^−1^ FW)	Carotenoids (µg/g FW)
Control	0.93 ± 0.02d	1.02 ± 0.06d	0.97 ± 0.04e	1.98 ± 0.18cd	0.71 ± 0.05cd
NaCl	0.46 ± 0.06e	0.41 ± 0.06f	0.43 ± 0.24f	0.84 ± 0.40f	0.67 ± 0.13d
Se-1	2.63 ± 0.31b	2.21 ± 0.56b	2.42 ± 0.21b	2.40 ± 0.34b	0.20 ± 0.01e
Se-2	3.22 ± 0.28a	2.94 ± 0.49a	2.58 ± 0.14a	4.84 ± 0.21a	1.13 ± 0.22ab
Se-3	1.63 ± 0.40cd	1.19 ± 0.11c	1.41 ± 0.22d	1.53 ± 0.20e	1.22 ± 0.20a
NaCl+Se-1	1.51 ± 0.23cd	1.20 ± 0.24c	1.36 ± 0.15d	1.51 ± 0.30d	0.14 ± 0.01f
NaCl+Se-2	2.04 ± 0.34c	1.72 ± 0.39bc	1.89 ± 0.16c	1.92 ± 0.45c	1.14 ± 0.22ab
NaCl+Se-3	0.98 ± 0.03d	0.89 ± 0.06e	0.94 ± 0.04e	1.46 ± 0.16d	0.86 ± 0.21c

Data presented are the mean ± SD of five replications. Different letters denote significant differences between treatments at p ≤ 0.05. NaCl = 200 mM NaCl; Se-1 = 75 μmo L^−1^ Se; Se-2 = 100 μmo L^−1^ Se; Se-3 = 125 μmo L^−1^ Se.

FW, fresh weight.

### Total soluble sugar, protein content, and α-amylase activity

With Se priming, significantly higher TSS and TSP contents up to 48% and 41%, respectively, were recorded compared to control seedlings grown without NaCl stress (**Table 2**). In addition, Se seed priming affected the TSS and TSP by 36% and 43%, respectively, under salinity stress. In particular, Se-2 improved the TSS and TSP contents by 48% and 45%, respectively, as opposed to NaCl-treated seedlings alone. The data from the current study depicted 75% higher activity of the α‐amylase enzyme in Se-pretreated seedlings compared to non-Se-primed seedlings. The interactive effects of Se and NaCl resulted in a 33% increase in α-amylase activity compared to the control condition. Statistical analysis revealed that Se-2-treated plants significantly exhibited maximum α-amylase activity and soluble sugar content (33% and 21%, respectively) under both stressed and non-stressed conditions (**Table 3**).

**Table 3:** Influence of Se pretreatment on total soluble sugar, α-amylase, and nutrient content of *Brassica rapa* seedlings under saline conditions.

Treatments	α-Amylase (U g^−1^ FW)	Total soluble sugar (mg g^−1^ FW)	Selenium (mg g^−1^ DW)	Na^+^ (mg g^−1^ DW)
Control	4.51 ± 1.11b	1.68 ± 0.22d	0.41 ± 0.04c	12.81 ± 0.31de
NaCl	1.45 ± 0.12e	0.82 ± 0.05f	0.09 ± 0.12d	28.15 ± 0.41a
Se-1	3.6 ± 0.54c	3.48 ± 0.21b	0.85 ± 0.06a	8.34 ± 0.42ef
Se-2	5.98 ± 0.45a	4.26 ± 0.05a	0.96 ± 0.02a	6.42 ± 0.32f
Se-3	4.11 ± 0.14bc	2.42 ± 0.34c	0.74 ± 0.06ab	9.13 ± 1.00e
NaCl+Se-1	2.34 ± 0.55d	1.66 ± 0.26d	0.57 ± 0.14bc	21.36 ± 1.31b
NaCl+Se-2	4.34 ± 1.01bc	2.24 ± 0.57c	0.61 ± 0.02bc	13.48 ± 0.60d
NaCl+Se-3	3.23 ± 0.21c	1.22 ± 0.21e	0.48 ± 0.05c	14.68 ± 0.97c

Data presented are the mean ± SD of five replications. Different letters denote significant differences between treatments at p ≤ 0.05. NaCl = 200 mM NaCl; Se-1 = 75 μmo L^−1^ Se; Se-2 = 100 μmo L^−1^ Se; Se-3 = 125 μmo L^−1^ Se.

FW, fresh weight; DW, dry weight.

### Proline content

**Figure 1:**
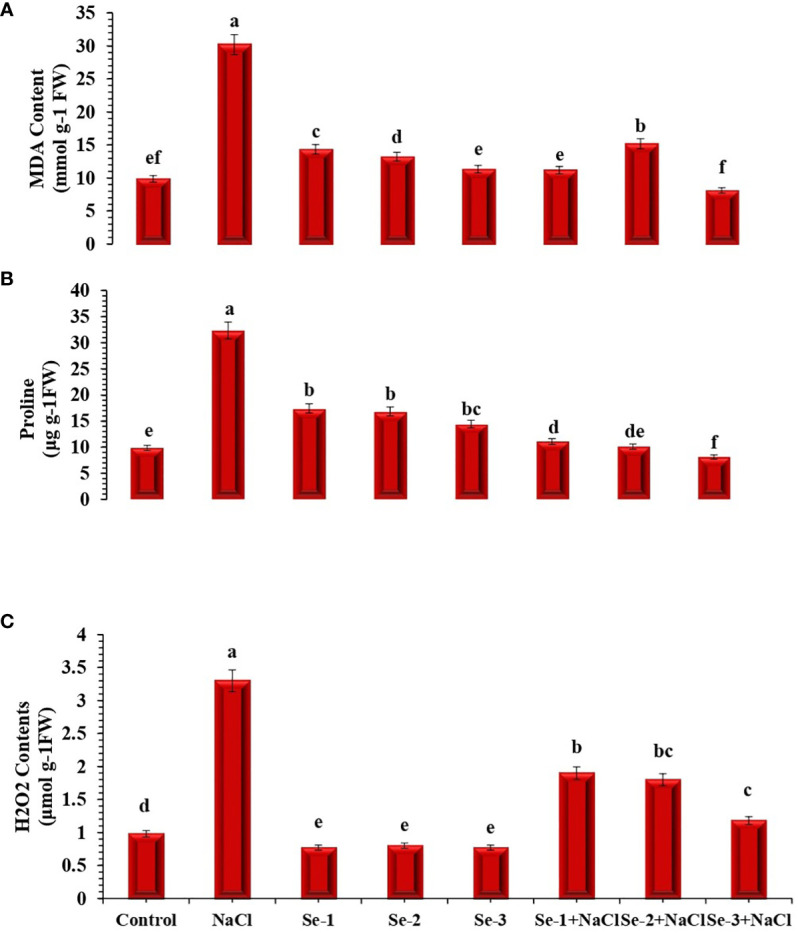
Influence of Se pretreatment on the non-enzymatic content of *Brassica rapa* seedlings under saline conditions. **(A)** Malondialdehyde (MDA). **(B)** Proline. **(C)** Hydrogen peroxide (H_2_O_2_). Data presented are the mean ± SD of five replications. *Different letters* denote significant differences between treatments at *p* ≤ 0.05. *C*, control. *NaCl* = 200 mM NaCl; *Se-1* = 75 μmo L^−1^ Se; *Se-2* = 100 μmo L^−1^ Se; *Se-3* = 125 μmo L^−1^ Se.

Salinity stress enhanced the biosynthesis of proline by 21% (**Figure 1**). Additional proline was synthesized in salt-stressed plants that received Se-2 pretreatment; generally, Se-2-treated seedlings exhibited 14% more proline content than those grown in the normal condition (**Figure 1**). The effect of Se treatment under the stress condition was observed between Se-2 and NaCl for proline synthesis in treated seedlings, which enhanced the level of proline in treated seedlings by 13% compared to the control condition.

### Effect of Se on the H_2_O_2_ and MDA levels

Salt stress enhanced the H_2_O_2_ and MDA contents in *B. rapa* seedlings by 29% and 33%, respectively, compared to the control conditions. However, under salinity regimes, lower levels of H_2_O_2_ and MDA were recorded in Se-treated seedlings in the order Se-2, Se-1, and Se-3 (**Figure 1**). An antagonistic effect was observed between Se-2 and NaCl regarding the biosynthesis of H_2_O_2_ and MDA, which decreased by 48% and 49%, respectively, compared to the normal condition (**Figure 1**). A significantly reduced level of MDA was depicted by Se-2-pretreated seedlings compared to control seedlings under salt stress conditions.

### Se-priming enhanced the Se content by reducing the Na uptake

Salt toxicity significantly decreased the uptake of Se by 21% compared to the control condition. Nevertheless, improved plant nutrition was observed in Se-primed seedlings in all conditions compared to salt-stressed seedlings not treated with Se. The current research revealed that Se-2 was the most effective treatment regarding Se uptake, which increased Se by 13% and reduced Na uptake by 17% in both the control and salinity-stressed *B. rapa* plants. Moreover, Se-2 significantly enhanced the uptake of Se in both stressed and non-stressed conditions by 11% and 14%, respectively, compared to the control condition (**Table 3**).

### Activity of antioxidant enzymes

**Figure 2:**
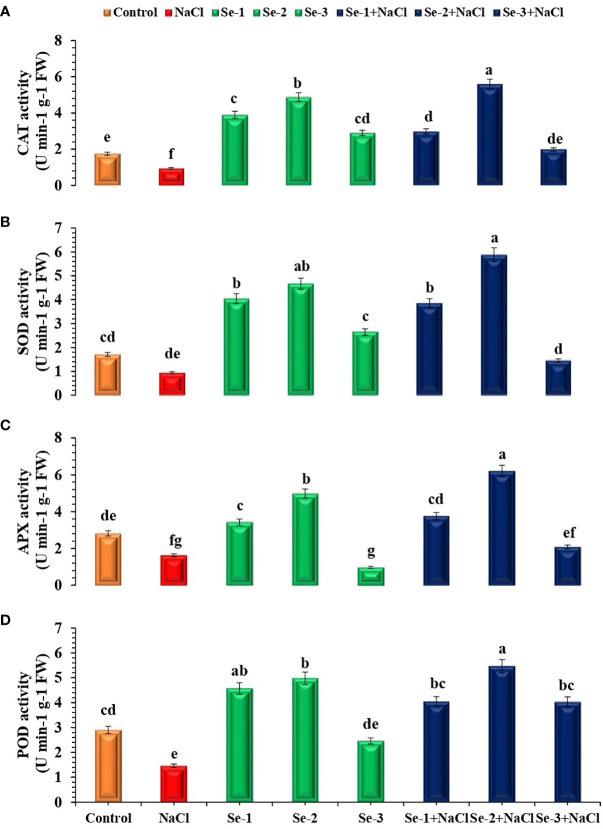
Influence of Se pretreatment on the antioxidant activity of *Brassica rapa* seedlings under saline conditions. **(A)** Catalase (CAT). **(B)** Superoxide dismutase (SOD). **(C)** Ascorbate peroxidase (APX). **(D)** Peroxidase (POD). Data presented are the mean ± SD of five replications. *Different letters* denote significant differences between treatments at *p* ≤ 0.05. *C*, control. *NaCl* = 200 mM NaCl; *Se-1* = 75 μmo L^−1^ Se; *Se-2* = 100 μmo L^−1^ Se; *Se-3* = 125 μmo L^−1^ Se.

Higher SOD activity was observed in Se-2-treated *B. rapa* seedlings under both stressed and non-stressed conditions, which increased by 29% and 19% as compared to the control. When subjected to salinity stress, the Se-2-treated seedlings revealed an elevated SOD activity compared to the control under both stressed and non-stressed conditions (20% and 23%, respectively) (**Figure 2**). Se priming enhanced the ascorbate peroxidase (APX) activity in treated seedlings by 26% compared to normal conditions. Moreover, Se-2-pretreated seedlings showed 30% and 24% augmented APX activity under stressed and non-stressed conditions, respectively, compared to the control (**Figure 2**). Priming with Se increased the activity of CAT by 35% and that of POD by 43% compared to the control condition. Statistical analysis revealed that Se-2-primed seeds exhibited 16% and 35% elevation of the activity of CAT in both stressed and non-stressed conditions, respectively, compared to the control condition. The same pattern was observed for POD in Se-treated seedlings grown in stressed and non-stressed conditions (**Figure 2**). In this case, Se-2-treated non-stressed and stressed seedlings demonstrated 34% and 18% higher POD activity, respectively, compared to the control condition.

### Expression of antioxidant genes

**Figure 3:**
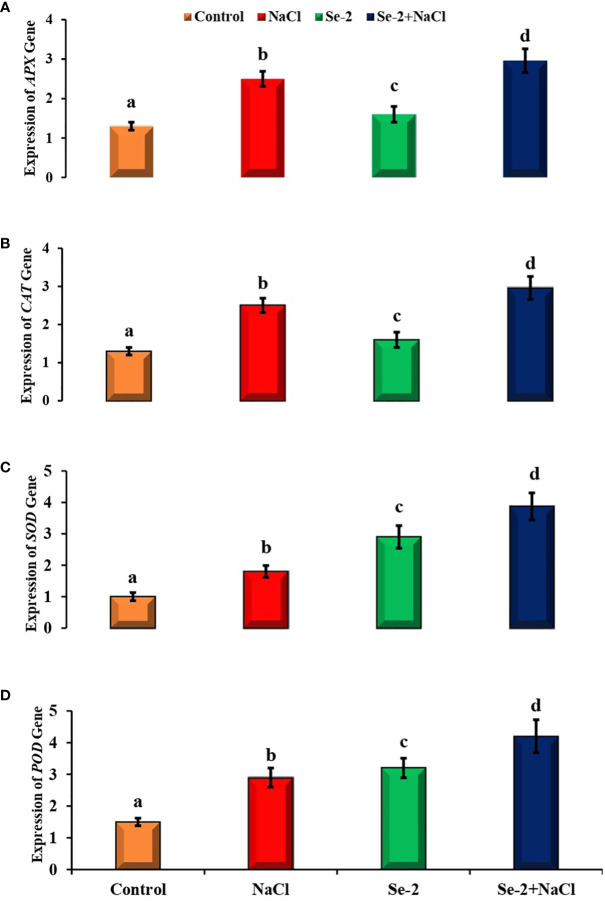
Quantitative real-time PCR analysis of the transcript levels of antioxidant defense-related genes in the shoots of turnip plants treated with Se and NaCl. **(A–D)** Relative expression of the *APX* **(A)**, *CAT* **(B)**, *SOD* **(C)**, and *POD* **(D)** genes. *Bars* represent the mean values of three independent experiments with standard errors. *Different letters* denote significant differences between treatments (*p* ≤ 0.05). *C*, control. *NaCl* = 200 mM NaCl; *Se-2* = 100 μmo L^−1^ Se.

Salinity and Se-2 treatment modulated the expression levels of the stress-responsive genes compared to the control condition. The transcript level of the *APX* gene was enhanced by about 1.6-fold with the administration of Se-2 to salt-stressed *B. rapa* plants. Salt-stressed plants that received Se-2 treatment exhibited a 2.96-fold higher expression of *APX* than that of control plants under saline conditions (**Figure 3**). The expression level of *CAT* was elevated by 4.2-fold in salt-stressed *B. rapa* plants compared to the control. Similarly, the transcript level of *CAT* increased by 3.98-fold in Se-2-treated plants subjected to salinity stress. Salt-treated plants demonstrated a 1.8-fold higher transcript level of *SOD* compared to the control. Similarly, the application of Se-2 upregulated the expression of *SOD* by 3.87-fold compared to control plants under saline regimes. It was also observed that the transcript level of *POD* was elevated by 2.9-fold when *B. rapa* plants were grown under salt stress compared to the control. Additionally, there was a 4.2-fold increase in gene expression in Se-2-treated plants under salt stress.

## Discussion

Appropriate seed germination is one of the primary initial growth phases that determine the future growth, stand, and yield of crop plants, especially under biotic and abiotic stresses. The data of the current study revealed that salt stress significantly reduced the germination percentage and early seedling growth of *B. rapa*. Several other studies have demonstrated the harmful effects of salinity stress on seed germination and the growth of seedlings (Gamalero and Glick, 2022). Selenium is not regarded as a fundamental nutrient because minute concentrations of this element may have harmful or beneficial effects on the growth of different plant species (Ismael et al., 2019; Hasanuzzaman et al., 2020; Buturi et al., 2021). In this study, the application of Se to salt-stressed *B. rapa* through seed priming elucidated the versatile features of this element. Data from the present study showed that salinity severely affected seed germination. Similar results of salt stress on the seed germination of other plants have been reported by various researchers (Egamberdieva et al., 2019). However, in the current study, different concentrations of Se showed multifaceted results under optimal and stressed conditions. It was found that seed pretreatment with Se-2 solution (100 μmo L^−1^) was more effective in alleviating salt stress and enhancing seed germination and seedling growth compared to the other two priming concentrations. A lower concentration of Se used for seed priming enhanced seed germination and seedling growth (Moulick et al., 2018). However, Se toxicity at a higher concentration induced negative effects, resulting in a decrease in the growth parameters (Çatav et al., 2022). In this study, the lower Se concentration (Se-1, 75 μmo L^−1^) decreased salinity stress and improved growth under both stressed and non-stressed conditions (**Table 1**). The higher Se concentration (Se-2, 100 μmo L^−1^) alleviated NaCl stress by improving the germination index, seedling length, and fresh weight in stressed plants; however, Se induced toxicity at the highest concentration (Se-3, 100 μmo L^−1^), which decreased the germination index, seedling length, and fresh weight in non-stressed plants (**Table 1**). A lot of researchers have reported the interactive effects of Se and salt stress in hydroponic conditions. The results of these studies showed that the adverse effects of salt stress on the germination and growth of seedlings may be mitigated by seed priming with Se. Nevertheless, there is a dearth of knowledge describing the potential of seed pretreatment with various Se concentrations in alleviating salt stress in vegetable crops (**Table 1**). The experimental study results demonstrated the beneficial role of Se seed priming in the alleviation of salt stress, and we advocate using this strategy to prevail over salinity issues in arable areas.

The photo-oxidation reactivity triggered by salinity increased the biosynthesis of superoxide radicals and hydrogen peroxide (H_2_O_2_). The augmented synthesis of superoxide radicals and H_2_O_2_ decreased the chloroplast and thylakoid membranes, resulting in the degradation of the photosynthetic pigments, including Chl *a*, Chl *b*, carotenoids, and total chlorophyll (Rejeb, 2015). Additionally, the elevated activity of chlorophyllase and the cellular injuries under saline conditions caused chlorophyll deprivation and reduced the water content in plants (Aazami et al., 2021; Giordano et al., 2021). In the present research, the higher concentration of chlorophyll in Se-2-treated seedlings suggests the efficacy of Se in alleviating the degradation of Chl *a*, Chl *b*, carotenoids, and total chlorophyll by reducing the negative impacts of salt stress (**Table 2**). Earlier reports also illustrated that exogenously applied Se decreased the degradation of photosynthetic pigments in crops subjected to abiotic stresses (Andrade et al., 2018; Amin et al., 2020; Farooq et al., 2020).

The findings of the present research exhibited improvements in the production of total soluble proteins and in the sugar content of Se-pretreated seedlings. The higher protein level in halotolerant plants under saline conditions reduces cellular damage and enables these plants to sustain membrane integrity (Singh and Jha, 2017). Selenium alters the metabolic activities of amino acids to augment protein synthesis by replacing the sulfur ions in sulfur, including amino acids such as cysteine and methionine (Ahmad et al., 2017; Yilmaz et al., 2018). Hence, it may be assumed that the modulated metabolism in Se-2-treated salt-stressed seedlings encouraged higher protein synthesis. Parallel to the results of the current study, Põldma et al. (2013) found an amplified concentration of total soluble protein in Se-treated plants subjected to salt stress. The results of the study showed that NaCl reduced the biosynthesis of TSS in stressed seedlings (**Table 2**), whereas Se pretreatment enhanced the synthesis of TSS. Nasibi et al. (2022) revealed that soluble sugars reduce the membrane osmotic potential and decrease water loss by binding to the membrane lipid bilayer. Sugars form hydrogen bonds with polar polypeptide residues and avert protein deprivation (Zhang et al., 2021). The increased concentration of TSS in Se-treated seedlings decreased cellular damage by regulating a balance among the cellular vacuole, osmotic cytosol intensity, and external medium that uphold cell turgor (Kaur et al., 2021; Kumari et al., 2021). Our results also demonstrated higher activity of α-amylase and soluble sugar content in Se-primed seedlings (**Table 3**).

Plants enhance the synthesis of stress-responsive proline to decrease protein degradation, detoxify the augmented level of ROS, and maintain membrane integrity (Mohammadi and Roshandel, 2020). Several studies have confirmed the stress alleviation efficiency of this low-molecular-weight organic solute (Zhu et al., 2020). The results of the current study also showed higher proline accumulation in NaCl-stressed seedlings (**Figure 1**). Selenium application further enhanced proline accumulation in treated seedlings under stress. Stress alleviation through higher proline synthesis in Se-treated salted plants has been observed by El Moukhtari et al. (2020) and Umair et al. (2020) in *Phaseolus vulgaris* and *Breasica oleracae* crops, respectively. Proline is a stress biomarker that acts as an osmolyte to maintain cellular integrity and scavenge ROS (Kaur et al., 2021). Proteolysis and conservation of the proline precursors enhance the level of this osmoprotectant in plants subjected to salt stress (Kaur et al., 2021). The increased level of proline helps in the reduction of ROS and MDA levels in salt-stressed plants (Zhang et al., 2021). The higher Se content in Se-treated seedlings may have accomplished some basic functions of proline regarding the alleviation of salt stress. Ibrahim (2014) also found decreased proline content in Se-pretreated wheat plants under drought stress.

Salt stress enhances the biosynthesis of H_2_O_2_, which is the foundation of cellular contraction, apoptosis, DNA disintegration, and chromatin abridgment (Jeong et al., 2022). Enzymatic and non-enzymatic antioxidants assist in stress mitigation by scavenging the higher level of H_2_O_2_ (Danouche et al., 2020). In this study, Se-2 seed priming significantly decreased the levels of H_2_O_2_ in treated plants (**Figure 1**). The study results concerning the lower accumulation of H_2_O_2_ in Se-treated salt-stressed seedlings are in agreement with the findings of Rady et al. (2021) and Rehman et al. (2022). Similarly, salt-stressed *B. rapa* seedlings showed a higher MDA content than the control. Conversely, Se-2 pretreatment significantly lowered the MDA content in NaCl-stressed plants (**Figure 1**). The seedlings developed from Se-2-primed seeds exhibited decreased MDA levels compared to the non-Se-treated seedlings under salt stress, signifying the function of Se in reducing lipid peroxidation through improving the antioxidant system, which safeguarded the membrane integrity (Li et al., 2019; Soleymanzadeh et al., 2020). The present findings are also validated by other studies that showed that exogenous Se application decreased the MDA levels in other plant species under stress (Huang et al., 2018; Ulhassan et al., 2019).

The Na^+^ ion content was lower in Se-primed seedlings compared to non-primed seedlings. It is assumed that a similar transporter is shared by Se and Na. Hence, the antagonistic relationship between Se and Na may have reduced the translocation of Na ions. Selenium reduced the Na^+^ content in treated seedlings through binding it to the cell wall of the root (Habibi, 2017). The cysteine synthase enzyme converts the inorganic Se absorbed by the aleurone layer of seeds into organic Se (Hu et al., 2022). The results of this study revealed that the lower concentration of Se (75 μmo L^−1^) effectively decreased the Na content in salt-stressed plants (**Table 3**). Some early studies have shown that the application of a lower concentration of inorganic Se easily converts into the organic forms of Se, such as SeMet and selenomethyl cysteine, which have antioxidant capacity and can alleviate plant stress (Wrobel et al., 2020; Gui et al., 2022).

Salt-induced oxidative stress augments ROS biosynthesis and impedes the root growth, shoot growth, and biomass production of plants. Antioxidant enzymes, including SOD, APX, CAT, and POD, alleviate oxidative damage by scavenging the higher level of ROS (Jahan et al., 2019; Moustafa-Farag et al., 2020). These enzymes protect plants from ROS-induced plasma membrane peroxidation (Sharma et al., 2020). The current study showed elevated SOD, APX, CAT, and POD activities in Se-2-treated salt-stressed seedlings. The findings of the current research are compatible with preceding studies showing that exogenously supplemented Se improved the activity of SOD, APX, CAT, and POD in salt-stressed seedlings (Sheikhalipour et al., 2021; Jabeen et al., 2021). Higher enzyme activity may have detoxified free radicals and decreased ROS-induced oxidative injury (**Figure 2**). El-Badri et al. (2022a) observed that exogenously applied Se upregulated the expression of stress-responsive antioxidant genes to mitigate salt stress in *B. napus* seedlings. Equally, Rossatto et al. (2017) reported that reverse transcription quantitative PCR (RT-qPCR) revealed higher expression levels of *CAT*, *APX*, and *SOD*, which detoxified ROS in rice plants to alleviate NaCl toxicity. Moreover, the data of the present research regarding the modulation of the expression levels of *POD*, *CAT*, *APX*, and *SOD* are in agreement with the findings of El-Badri et al. (2022b). They reported that modulations in the transcript levels of these antioxidant genes enhanced the activity of the respective antioxidant enzymes to induce salt tolerance by scavenging ROS in *B. napus* plants. The higher activity of these antioxidant enzymes converted H_2_O_2_ to H_2_O. Hence, the higher water content observed in Se-2-treated seedlings may be a result of the upregulated activity and higher expression levels of these enzymes (Yaldiz and Camlica, 2021).

## Conclusion

It may be concluded that Se pretreatment through seed priming at concentrations of 75 μmo L^−1^, 100 μmo L^−1^, and 125 μmo L^−1^ Se reduced the toxic effects of salinity stress associated with seed germination and seedling growth of *B. rapa* by reducing the Na uptake and improving the Se uptake, suppressing the oxidative damage, and by improving the antioxidant system and the expression levels of the genes involved in the antioxidant system of plants. Taken as a whole, this work provides insights toward our understanding and discovery of the specific morphophysiological and biochemical mechanisms behind Se-induced salinity stress tolerance in *B. rapa*. Conversely, additional research work is compulsory to determine the transcriptomic, metabolomics, genomics, and proteomics inflections related to salt stress alleviation in Se-treated *B. rapa* plants under field conditions.

## Data availability statement

The original contributions presented in the study are included in the article/supplementary material. Further inquiries can be directed to the corresponding authors.

## Author contributions

NY and SA conceived the idea for this study. SH and WA performed the experiments. SA, NY,GL and SH prepared the manuscript draft. SA,GL and NY reviewed and edited the final version. All authors contributed to the article and approved the submitted version.
